# Multi-Omics Analysis Identifies SlLhcb13 as a Key Regulator of Tomato Resistance to *Botrytis cinerea*

**DOI:** 10.3390/plants15091360

**Published:** 2026-04-29

**Authors:** Dan Luo, Xiaojie Peng, Weiqiang Yan, Yujin Wang, Ke Liu, Lixia Li, Zhe Wu, Hongmei Nie, Sheng Sun, Wenhui Sun, Jun Cai

**Affiliations:** 1 Shanxi Key Laboratory of Germplasm Resources Innovation and Utilization of Vegetable and Flower, College of Horticulture, Shanxi Agricultural University, Jinzhong 030801, China; 2 National Key Laboratory for Germplasm Innovation and Utilization of Horticultural Crops, Huazhong Agriculture University, Wuhan 430070, China

**Keywords:** multi-omics, *Botrytis cinerea*, plant immunity, photosynthesis, *SlLhcb13*

## Abstract

Gray mold caused by *Botrytis cinerea* poses a severe threat to tomato production. In this study, physiological, biochemical, transcriptomic, and proteomic analyses were integrated to characterize the dynamic responses of tomato ‘Ailsa Craig’ to *B. cinerea* infection. During *B. cinerea* infection, peroxidase (POD) activity showed a progressive increase, while catalase (CAT) activity was significantly upregulated at 24 hpi and remained stable through 48 hpi. Malondialdehyde (MDA) and hydrogen peroxide (H_2_O_2_) contents showed a delayed response, increasing significantly only at 48 hpi, whereas SOD activity exhibited a biphasic pattern. Transcriptome and proteome profiling identified 5824 differentially expressed genes and 124 differentially expressed proteins. Functional enrichment analysis highlighted defense-related pathways, including plant–pathogen interaction, flavonoid biosynthesis, and inositol phosphate metabolism. Notably, the chlorophyll a/b-binding protein *SlLhcb13* exhibited post-transcriptional upregulation despite transcriptional suppression. Functional validation demonstrated that overexpression of *SlLhcb13* enhanced resistance, whereas silencing increased susceptibility. These findings identify *SlLhcb13* as a positive regulator linking photosynthesis to immunity and provide new insights into the defense mechanisms of tomato.

## 1. Introduction

Tomato is one of the world’s major vegetable crops and is rich in vitamin C and lycopene, conferring high economic and nutritional value [1]. However, from pre-harvest production in the field to post-harvest cold-chain logistics, *Botrytis cinerea* remains a persistent threat throughout the entire tomato production and supply chain, causing substantial economic losses [2]. Gray mold, a destructive disease caused by *B. cinerea*, initially appears as water-soaked lesions on leaves; as the disease progresses, these lesions gradually expand, soften, and decay, eventually becoming covered with a gray mold layer, which leads to leaf abscission and stem breakage. Disease incidence can reach 30–40% during the seedling stage and exceed 60% during the mature stage, with severe cases resulting in complete loss of photosynthetic capacity [3]. *B. cinerea* is a widely distributed necrotrophic fungus, and its pathogenicity is influenced by factors such as relative humidity and temperature; under conditions of high humidity and low temperature, the fungus reproduces rapidly, readily causing systemic infection in greenhouse-grown tomatoes, seriously impairing plant growth and fruit development and thereby reducing yield and quality [4]. The pathogenic mechanism of *B. cinerea* is dynamic and varies according to different infection strategies, enabling the fungus to survive under both favorable and unfavorable conditions [5]. *B. cinerea* invades the host by forming infection structures and releasing plant cell wall-degrading enzymes (PCWDEs) and other pathogenic factors [6]; under suitable environmental conditions, its spores germinate on the plant surface to produce germ tubes, after which the pathogen at the tips of these germ tubes penetrates host tissues or cells, ultimately establishing infection [7]. Studies have shown that the growth, development, and virulence of *B. cinerea* are closely associated with reactive oxygen species (ROS) and extracellular secreted proteins, and the pathogen can infect nearly all plant parts before and after harvest, making prevention and control extremely difficult [5]. Therefore, a comprehensive understanding of tomato response mechanisms to gray mold is of great significance for improving gray mold management.

During the early stage of gray mold infection, plants rapidly generate large amounts of ROS, thereby activating defense signaling networks. However, excessive ROS accumulation can also inflict oxidative damage on plant cells, and malondialdehyde (MDA) is widely recognized as an important indicator of membrane lipid peroxidation [8,9,10]. To maintain intracellular ROS homeostasis, plants activate an ROS-scavenging enzyme system mainly composed of superoxide dismutase (SOD) and peroxidase (POD), which alleviates stress through both independent and coordinated actions [11]. Under pathogen challenge, plants also accumulate osmotic adjustment substances such as soluble sugars and soluble proteins. These compounds not only protect cell membranes and proteins from damage and help maintain normal physiological activities, but also function as ROS scavengers to reduce oxidative injury, thereby reflecting the metabolic status of the host and the intensity of physiological and biochemical responses during plant–pathogen interactions [12].

To defend against pathogen invasion, plants have evolved a sophisticated innate immune system. When pathogens breach the plant cell wall, they activate pattern-triggered immunity (PTI) through pattern recognition receptors (PRRs) located on the cell membrane, thereby restricting pathogen invasion and proliferation [13,14,15,16,17]. During the co-evolution of plants and microorganisms, pathogens secrete effector molecules and other toxic virulence factors that interfere with and suppress the PTI response, thereby inducing effector-triggered susceptibility (ETS) and promoting infection [18]. In turn, plants have evolved effector-triggered immunity (ETI) as a second layer of defense. Under natural selection, pathogens may suppress ETI by escaping recognition or by producing novel effectors, which in turn drives plants to evolve new resistance proteins and reactivate their defense systems. This evolutionary arms race persists continuously and ultimately results in dynamic coexistence [19,20].

In plant–pathogen interactions, photosynthesis is not only highly vulnerable to pathogen attack but also serves as a central hub for the regulation of plant defense responses. As the fundamental metabolic process in green plants, photosynthesis consists of light-dependent reactions mediated by two photosystems, Photosystem I (PSI) and Photosystem II (PSII) [21,22,23,24]. As the initial electron donor in the photosynthetic electron transport chain, PSII catalyzes water splitting through the absorption of light energy [25,26]. Within the intricate structure of PSII, light-harvesting complex II (LHCII) proteins encoded by the *Lhcb* gene family play indispensable roles. As the peripheral antenna system of PSII, LHCII is composed of multiple transmembrane proteins that efficiently bind photosynthetic pigments, including chlorophyll a/b, xanthophylls, and carotenoids [27,28]. The *Lhcb* gene family exhibits marked interspecific diversity, including variation in gene copy number as well as species-specific expression patterns and physiological functions [29,30].

Accumulating evidence suggests that under abiotic stresses such as high light or wounding, the chlorophyll-binding proteins *Lhcb6* and *Lhcb5* play distinct roles in development, thylakoid architecture, and PSII photoprotection in *Physcomitrella patens* [31]. In *Arabidopsis thaliana*, the stress-induced paralog of *Lhcb4*, *Lhcb8* (*Lhcb4.3*), regulates adaptive responses to long-term abiotic stress by restructuring the functional organization of PSII [32]. When chloroplast function is disrupted by salt stress, RPOTp initiates retrograde signaling, revealing a possible compensatory mechanism triggered by chloroplast dysfunction [33]. In celery, *Lhcb1* is upregulated under stress conditions such as cold, heat, salinity, and drought [34]. In *A. thaliana*, the *Lhcb1-6* genes respond to stomatal movement and participate in ABA signaling, thereby influencing ROS homeostasis and enhancing plant stress tolerance [35,36]. Heterologous overexpression of *LeLhcb2* in tobacco enhances tolerance to low-temperature stress while reducing PSII photoinhibition [37]. Likewise, overexpression of *MdLhcb4.3* from apple not only increases chlorophyll content in transgenic *Arabidopsis* lines but also enhances tolerance to drought and osmotic stress, whereas knockout mutants of *AtLhcb6*, *AtLhcb5*, and *AtLhcb4* exhibit significantly reduced chlorophyll content [38]. The light-harvesting antenna protein encoded by *Lhcb-Ppn3* from peach belongs to the *Lhcb6* subfamily, and its expression decreases by 60% in leaves infected with leaf curl disease, suggesting that it may impair photosynthetic efficiency by affecting PSII stability [39,40]. Collectively, the Lhcb protein family exhibits functional diversity and species specificity in plant stress responses, serving both as direct protectors of the photosynthetic core machinery and as important hubs linking chloroplast and nuclear signaling pathways. However, whether members of the Lhcb protein family participate in the regulation of disease resistance during the tomato-*B. cinerea* interaction remains largely unclear.

Given the complexity of tomato resistance mechanisms against gray mold, this study aimed to systematically elucidate response patterns at both the transcriptional and translational levels through an integrated multi-omics strategy. To this end, tomato leaves collected at 0 and 24 h after *B. cinerea* inoculation were used as experimental materials, and key physiological indicators were assessed to verify stress responses. Subsequently, RNA-Seq and TMT-based proteomic analyses were employed to identify differentially expressed genes and proteins. Through bioinformatic analysis, we detected marked differences between transcriptional and translational regulation and accordingly selected several candidate genes associated with disease resistance. Among these, *SlLhcb13* attracted particular attention because of the contrasting patterns observed in its transcript and protein accumulation. To verify its function, we used transient transformation and confirmed that overexpression of *SlLhcb13* significantly alleviated disease symptoms, whereas silencing of this gene aggravated symptom development. This study not only highlights the importance of post-transcriptional regulation in tomato disease resistance responses but also identifies *SlLhcb13* as a novel positive regulator of disease resistance, thereby providing a foundation for future investigation of its mechanism of action and for molecular breeding aimed at improving disease resistance.

## 2. Results

### 2.1. Gray Mold Induces Oxidative Stress Response in Tomato Seedlings

**Figure 1:**
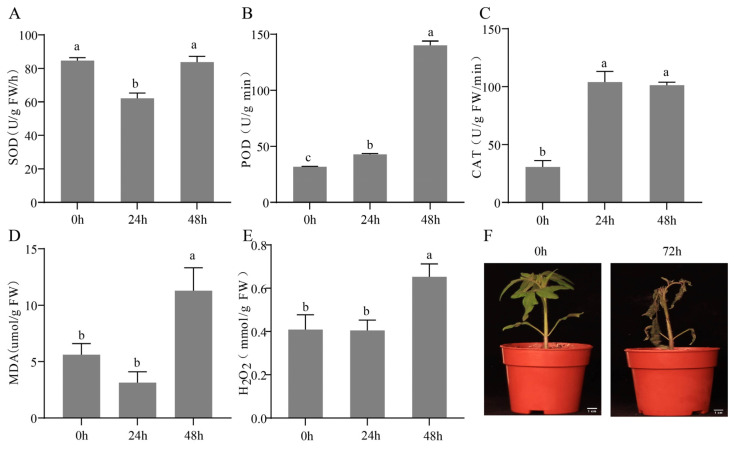
Effects of *B. cinerea* infection on tomato physiological indices and phenotypes. (**A**–**E**) Temporal dynamics of physiological and biochemical parameters in tomato leaves after infection with *B. cinerea* at 0 (control), 24, and 48 h, including the activities of superoxide dismutase (SOD), peroxidase (POD), and catalase (CAT), as well as the contents of malondialdehyde (MDA) and hydrogen peroxide (H_2_O_2_). (**F**) Representative disease symptoms of tomato plants at 0 h (control) and 72 h post-inoculation with *B. cinerea*. Data are presented as the mean ± standard deviation (*n* = 3). Statistical differences were assessed by one-way ANOVA followed by Duncan’s multiple range test. Different lowercase letters indicate significant differences (*p* < 0.05) among time points for the same parameter. Bar = 1 cm.

To evaluate the oxidative damage caused by *B. cinerea* in tomato seedlings, various physiological and biochemical parameters were measured at multiple time points after inoculation. At 24 h post-inoculation, SOD activity was significantly reduced compared with that in the 0 h control (Figure 1A), whereas POD and catalase (CAT) activities were significantly increased (Figure 1B,C). In contrast, the contents of MDA and hydrogen peroxide (H_2_O_2_) did not differ significantly between 0 h and 24 h (Figure 1D,E). However, at 48 h, SOD activity, POD activity, and the contents of MDA and H_2_O_2_ were all significantly increased relative to those at 24 h (Figure 1A,B,D,E), whereas CAT activity remained unchanged (Figure 1C). Overall, POD, CAT, MDA, and H_2_O_2_ showed a continuous upward trend throughout the infection period, whereas SOD activity displayed a biphasic pattern characterized by an initial decrease followed by an increase. These physiological changes were closely associated with the observed disease phenotypes. At 72 h post-inoculation, extensive brown lesions covered the leaves, and the plants exhibited pronounced wilting (Figure 1F), consistent with the severe oxidative damage indicated by the marked increases in MDA and H_2_O_2_ levels. Excessive accumulation of ROS caused oxidative damage in the plants; *B. cinerea* suppressed SOD activity while simultaneously aggravating membrane lipid peroxidation, thereby severely impairing normal tomato growth and development.

### 2.2. Identification and Functional Enrichment Analysis of Differentially Expressed Genes

**Figure 2:**
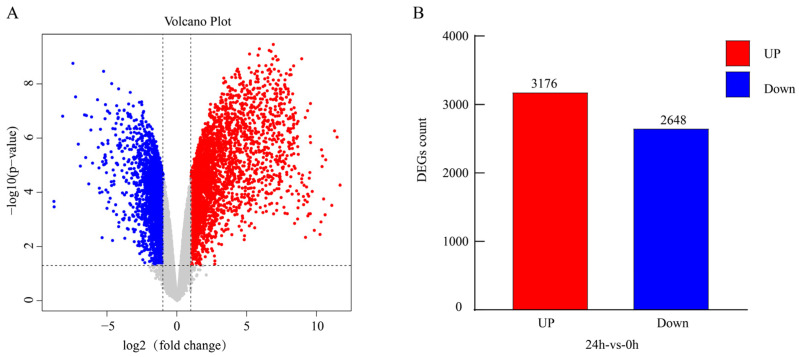
Identification of differentially expressed genes in tomato seedlings induced by *B. cinerea.* (**A**) Volcano plot showing red, blue, and gray dots representing significantly upregulated, significantly downregulated, and non-differentially expressed genes, respectively. The vertical dashed lines represent the fold change threshold (|log_2_FC| = 1), and the horizontal dashed line represents the significance threshold (FDR = 0.05). (**B**) Bar chart showing the numbers of upregulated and downregulated genes.

To identify genes in tomato seedlings that respond to *B. cinerea* infection, RNA-seq was performed on samples collected at 0 h (control) and 24 h post-inoculation. Compared with the 0 h control, a total of 5824 DEGs were identified, including 3176 upregulated genes and 2648 downregulated genes (Figure 2). DEGs were screened using the criteria |log_2_FC| > 1 and FDR < 0.05.

**Figure 3:**
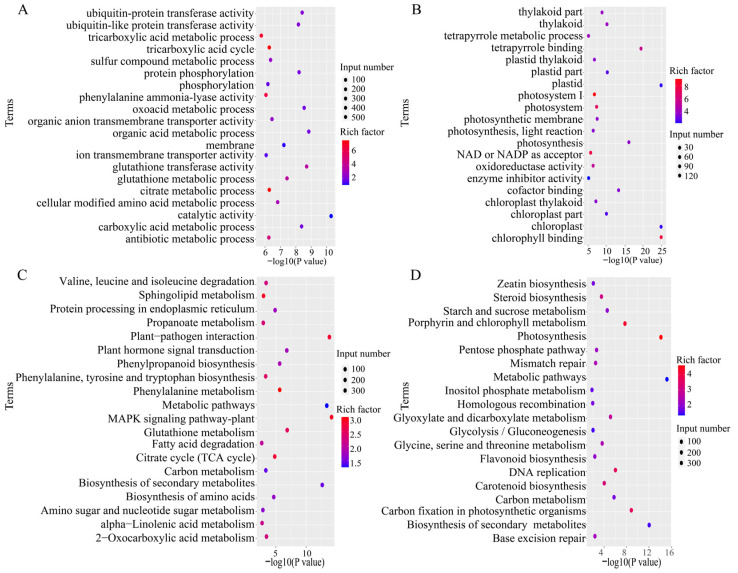
The top 20 GO entries and KEGG pathways enriched in gray mold stress-responsive genes. (**A**,**B**) The top 20 GO entries significantly enriched in upregulated and downregulated DEGs, respectively. (**C**,**D**) The top 20 KEGG pathways significantly enriched in upregulated and downregulated DEGs, respectively. In the figure, the horizontal axis represents the fold change in differential expression, and the vertical axis represents the name of the GO entry or KEGG pathway. The size of each bubble indicates the number of genes enriched in the corresponding function or pathway, and the color of the bubble reflects the significance of enrichment (*p*-value).

To further investigate the biological functions of DEGs involved in the tomato response to *B. cinerea*, we performed both Gene Ontology (GO) and Kyoto Encyclopedia of Genes and Genomes (KEGG) enrichment analyses. GO analysis showed that upregulated and downregulated DEGs displayed contrasting enrichment patterns. Specifically, upregulated genes were significantly enriched in catalytic activities associated with defense and stress responses, such as phenylalanine ammonia-lyase activity, ubiquitin-protein transferase activity, and glutathione transferase activity (Figure 3A). In contrast, downregulated genes were mainly enriched in biological processes and cellular components related to photosynthesis and energy metabolism, including chloroplast, photosynthesis, chlorophyll binding, and cofactor binding (Figure 3B).

To further explore the metabolic pathways associated with these DEGs, KEGG pathway analysis was performed. The results showed that pathways enriched in upregulated genes were closely associated with tomato resistance to pathogen invasion, mainly including plant–pathogen interaction, mitogen-activated protein kinase (MAPK) signaling pathway, glutathione metabolism, and biosynthesis of secondary metabolites (Figure 3C). In contrast, downregulated genes were significantly enriched in porphyrin and chlorophyll metabolism, photosynthesis, glyoxylate and dicarboxylate metabolism, carbon metabolism, carbon fixation in photosynthetic organisms, and biosynthesis of secondary metabolites (Figure 3D). Notably, the “biosynthesis of secondary metabolites” pathway was significantly enriched in both the upregulated and downregulated gene sets, suggesting that it may play a dual role in the complex regulatory network governing tomato responses to gray mold.

In summary, transcriptome analysis revealed extensive transcriptional reprogramming in tomato in response to *B. cinerea* infection. Functional enrichment analysis further indicated that this response displays the typical molecular characteristics of “enhanced defense and suppressed growth”. Specifically, genes associated with disease resistance and defense responses (such as phenylalanine metabolism and glutathione metabolism) and signal transduction (including plant–pathogen interaction and MAPK signaling pathway) were significantly upregulated, whereas genes involved in fundamental biological processes such as photosynthesis, energy metabolism, and carbon fixation were systematically downregulated. This coordinated shift in gene expression may reflect a strategic reallocation of energy resources in tomato, prioritizing defense responses under stress conditions.

### 2.3. Identification and Functional Annotation Analysis of Differentially Expressed Proteins in Tomato Responding to Gray Mold

**Figure 4:**
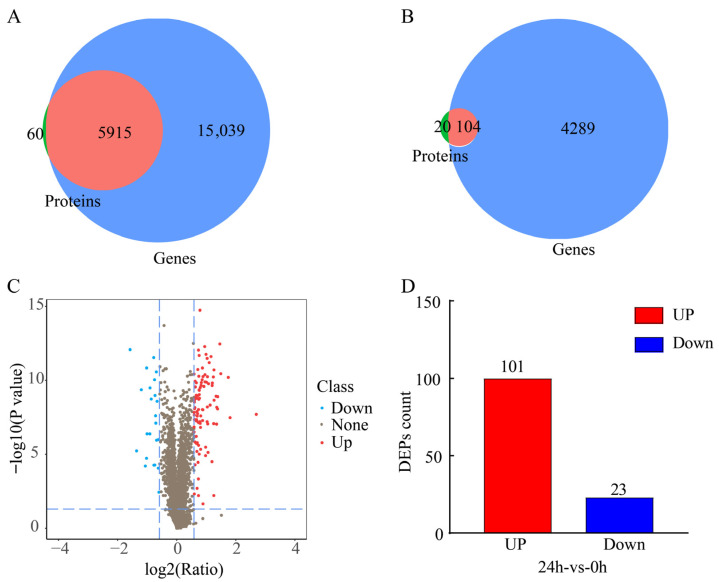
Identification of differentially expressed proteins in tomato under *B. cinerea.* (**A**) Venn diagram of all identified proteins and transcripts. (**B**) Venn diagram of differentially expressed proteins (DEPs) and differentially expressed genes (DEGs). (**C**) Volcano plot of differentially expressed proteins. The vertical dashed lines represent the fold change threshold (Fold Change = 1.5), and the horizontal dashed line represents the significance threshold (FDR = 0.05). (**D**) Distribution of the numbers of upregulated and downregulated differentially expressed proteins.

To investigate the response mechanism of tomato at the proteomic level, we performed quantitative proteomic analysis on tomato samples collected after 24 h of *B. cinerea* treatment (24 h) and from the control group (0 h). In total, 5975 proteins were identified (Figure 4A). Using the criteria of fold change > 1.5 and FDR < 0.05, we screened 124 DEPs (Figure 4B), including 101 upregulated proteins and 23 downregulated proteins (Figure 4C,D).

To clearly visualize the expression patterns of these DEPs and verify the reliability of the experiment, two-way clustering analysis was performed for all DEPs and samples. As shown in the heatmap in Supplementary Figure S1A, all biological replicates were distinctly clustered according to treatment condition: samples from the control group (0 h) clustered together, whereas samples from the treatment group (24 h) formed a separate cluster. This result indicates high consistency among samples within each group and clear differences between groups, confirming the strong reproducibility of the experiment and the reliability of sample grouping.

To further explore the biological functions of these DEPs, we first performed bioinformatics-based subcellular localization prediction. The results (see Supplementary Figure S1B) showed that the DEPs were predicted to be widely distributed throughout the cell, with the chloroplast (42 proteins) and cytoplasm (30 proteins) representing the two major compartments containing the highest numbers of localized proteins, followed by the plasma membrane (15), nucleus (14), and extracellular space (11). Notably, a large proportion of DEPs were predicted to localize to the chloroplast, suggesting a central role for this organelle in the response to *B. cinerea*. This prediction aligns well with the downregulation of photosynthesis-related genes observed in the transcriptome. On this basis, we further carried out GO, KOG, and KEGG enrichment analyses to systematically characterize the biological processes and signaling pathways represented by the DEPs.

**Figure 5:**
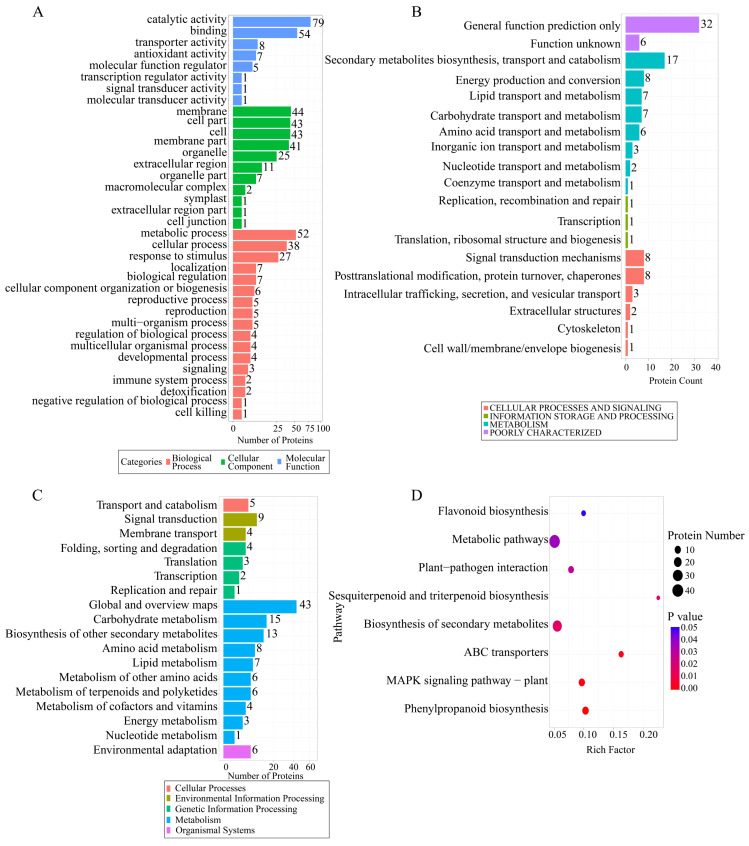
Functional annotation and pathway analysis of differentially expressed proteins. (**A**) GO enrichment analysis. (**B**) KOG functional classification histogram. (**C**) KEGG pathway enrichment histogram. In Figures (**A**–**C**), the height of the columns represents the number of enriched proteins. (**D**) DEPs pathway analysis. The *X*-axis is the enrichment factor, the *Y*-axis is the pathway; bubble size represents the total number of DEPs, and color represents significance.

To systematically characterize the functions of the DEPs, we performed GO, KOG, and KEGG functional enrichment analyses. GO analysis showed that the enriched functions of the DEPs displayed clear stress-response characteristics (Figure 5A). At the biological process level, DEPs were significantly enriched in response to stimulus, immune system process, detoxification, and cell killing, all of which are directly associated with pathogen defense. At the molecular function level, DEPs were mainly enriched in catalytic activity, binding activity, and signal transducer activity, indicating that tomato responds to *B. cinerea* infection by activating extensive biochemical reactions and signaling networks.

KOG analysis further supported this conclusion (Figure 5B). Proteins regulated by *B. cinerea* were widely involved in multiple core processes, mainly including the transport and metabolism of secondary metabolites, lipids, and carbohydrates, indicating that material and energy metabolism were extensively remodeled, as well as signal transduction mechanisms, post-translational modification, and protein turnover, revealing complex processes of signal perception and protein regulation. To assign these functions to specific metabolic pathways, we next performed KEGG pathway enrichment analysis. The results (Figure 5C,D) clearly pointed to the classical disease-resistance defense strategy of tomato. The most significantly enriched pathways included plant–pathogen interaction, plant MAPK signaling pathway, and ABC transporters, among other core defense-related signaling networks. Particularly noteworthy was the strong enrichment of secondary metabolic pathways such as phenylpropanoid biosynthesis and flavonoid biosynthesis, directly supporting the molecular mechanism by which tomato resists *B. cinerea* through the synthesis of antimicrobial secondary metabolites. In summary, the proteomic data collectively depict the defense landscape of tomato at multiple levels, namely, activation of core signaling pathways, reprogramming of metabolic flux, and synthesis of large amounts of antimicrobial secondary metabolites to establish a multilayered molecular network of disease resistance.

### 2.4. Association Analysis of Tomato Transcriptome and Proteome in Response to Gray Mold

**Figure 6:**
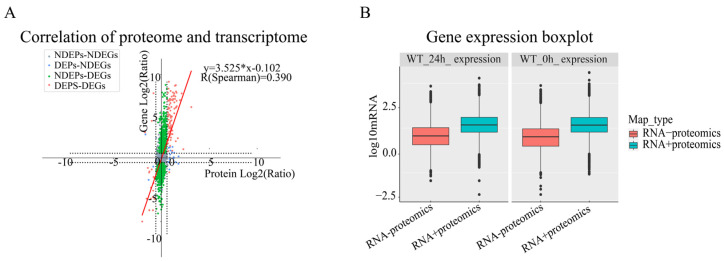
Overall association analysis of transcriptome and proteome data. (**A**) Scatter plot of transcript and protein abundance; the scatter plot shows the expression relationship of all gene-protein pairs. The *X*-axis is protein abundance, and the *Y*-axis is transcript abundance. The color of the points represents the differential expression status: gray (no difference), blue (only protein difference), green (only gene difference), red (both differences). The overall correlation coefficient (R) is marked in the figure. The vertical and horizontal dashed lines represent the baseline of no change (Log_2_ Ratio = 0) for proteins and transcripts, respectively. (**B**) Box plot of associated and unassociated gene expression levels; green and red represent genes associated with and not associated with the proteome, respectively.

To further explore the molecular regulatory network underlying the tomato response to gray mold, we conducted an integrated analysis of the transcriptome and proteome datasets. First, we evaluated the overall correlation between mRNA and protein expression. The results showed that the expression levels of all genes and proteins exhibited a weak to moderate correlation (*R* = 0.390, Figure 6A), indicating the presence of substantial post-transcriptional regulation. Interestingly, transcript levels of gene-protein pairs in the treatment group (24 h) showed greater dispersion, whereas those in the control group (0 h) were more consistent, suggesting that gray mold infection induced broader and more complex transcriptional regulation (Figure 6B). When we further focused on DEGs and DEPs, we found that DEG-DEP pairs showing the same expression trend displayed an increased correlation (*R* = 0.570), whereas pairs showing opposite expression trends exhibited a negative correlation (*R* = −0.200), further highlighting the importance of post-transcriptional regulation during stress responses (Supplementary Figure S2A–C).

**Figure 7:**
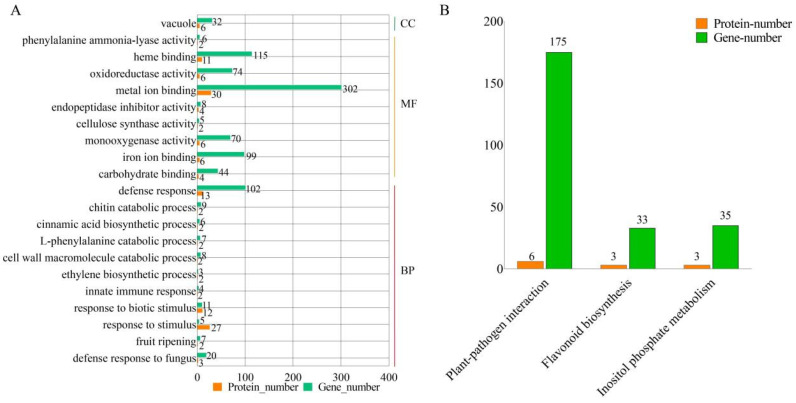
Enrichment association analysis of GO and KEGG for tomato core genes (DEG-DEP) responding to gray mold. (**A**) GO enrichment correlation analysis. (**B**) KEGG pathway enrichment correlation analysis. In (**A**,**B**), bar height represents the number of enriched proteins/genes in each pathway, with orange indicating protein counts and green indicating gene counts. The screening thresholds were FDR < 0.05 for transcripts and FDR < 0.1 for proteins.

To comprehensively dissect the multi-tiered regulatory network underlying tomato defense responses, we carried out an integrative multi-omics analysis. Differentially expressed genes (DEGs) were identified from transcriptomic data using the criteria of FDR < 0.05 and |log_2_FC| > 1, while differentially expressed proteins (DEPs) were identified from proteomic data using a slightly relaxed threshold of FDR < 0.1 and fold change > 1.5. Given that post-transcriptional regulation is common in plant immune responses and that proteomic detection has a relatively limited dynamic range, we adopted a moderately permissive statistical threshold at the protein level to capture more potential signals. Fold change was used as a secondary filter to maintain biological relevance. Subsequent functional enrichment analyses revealed that these molecules play central roles in tomato defense responses. GO enrichment analysis (Figure 7A) demonstrated significant enrichment in biological processes such as defense response, response to oxidative stress, and defense response to fungus. At the cellular component level, they were mainly associated with the vacuole. At the molecular function level, they were enriched in phenylalanine ammonia-lyase activity and oxidoreductase activity, suggesting that ROS scavenging and redox signaling are key functions. KEGG pathway analysis (Figure 7B) further linked these functions to specific defense networks, with the most significantly enriched pathways including plant–pathogen interaction, flavonoid biosynthesis, and inositol phosphate metabolism. Notably, plant–pathogen interaction and flavonoid biosynthesis are directly related to plant immune defense and the accumulation of secondary metabolites. The significant enrichment of inositol phosphate metabolism suggests active regulation of intracellular signal transduction networks, especially calcium and phospholipid signaling, during stress responses. In conclusion, this integrative analysis not only confirmed the complexity between transcript and protein expression but also identified a group of core defense genes that are co-regulated at both transcriptional and translational levels. The proteins encoded by these genes may localize to the vacuole to store antimicrobial compounds or regulate ion homeostasis. By activating key signaling pathways and promoting the synthesis of antimicrobial secondary metabolites, they together form an important molecular defense line for tomato against *B. cinerea*.

### 2.5. SlLhcb13 Positively Regulates Tomato Resistance to B. cinerea

**Figure 8:**
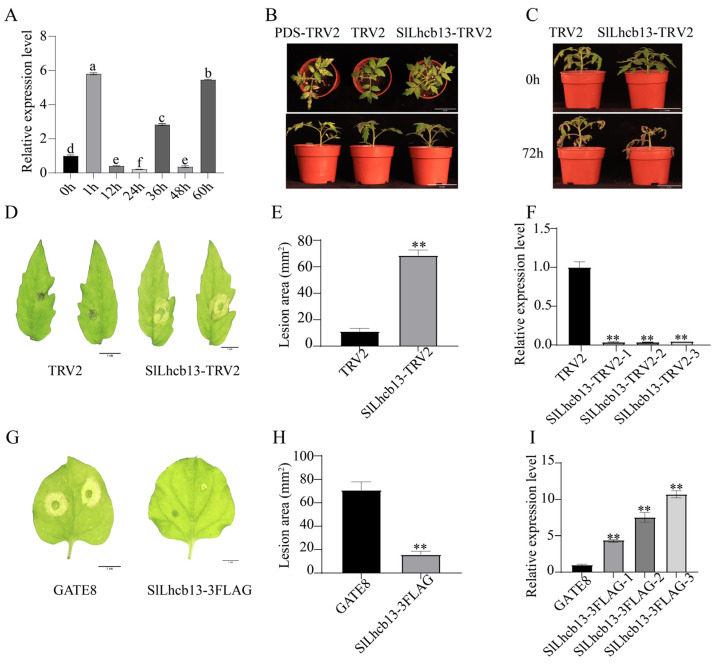
*SlLhcb13* positively regulates tomato resistance to *B. cinerea*. (**A**) Expression profile of *SlLhcb13* in wild-type plants inoculated with *B. cinerea* at indicated time points. Data are means ± SD (*n* = 3). Different letters denote significant differences (*p* < 0.05, one-way ANOVA with Duncan’s test). (**B**) Photobleaching phenotype of VIGS plants. Bar = 5 cm. (**C**–**E**) Disease symptoms (**C**,**D**) and lesion area (**E**) of *SlLhcb13*-silenced plants inoculated with *B. cinerea* for 72 h. ((**C**) Bar = 5 cm, (**D**) Bar = 1 cm.) (**G**,**H**) Disease symptoms (**G**) and lesion area (**H**) of *SlLhcb13*-overexpressing tobacco leaves inoculated with *B. cinerea* for 72 h. Bar = 1 cm. (**F**,**I**) Relative expression levels of *SlLhcb13* in silenced (**F**) and overexpressing (**I**) plants. Data are means ± SD (*n* = 3). * *p* < 0.05, ** *p* < 0.01 (Student’s *t*-test for (**E**,**H**); one-way ANOVA with Duncan’s test for (**F**,**I**)).

To identify key disease-resistance genes through integrated analysis, we focused on a candidate gene showing a distinctive regulatory pattern at the transcriptional and translational levels, Solyc12g011450.2 (designated *SlLhcb13*). This gene belongs to a conserved gene family (PLN00025), and its homologs in other species, such as *Arabidopsis* and rice, have been reported to enhance broad-spectrum disease resistance, making it a highly promising target for further study. To preliminarily verify its involvement in the tomato response to gray mold, we examined its expression pattern after pathogen inoculation by qRT-PCR. The results showed that the transcriptional expression of *SlLhcb13* was significantly induced at the early stage of *B. cinerea* infection, but was suppressed at 12 and 24 h post-inoculation (Figure 8A). Consequently, this gene was selected for further functional validation.

To determine the biological function of *SlLhcb13* in tomato resistance to *B. cinerea*, we generated overexpression and silencing materials using transient transformation technology. To further confirm its role, we also employed virus-induced gene silencing (VIGS) to reduce its endogenous expression. When the positive control plant PDS-TRV2 exhibited the expected photobleaching phenotype (Figure 8B), this indicated that the silencing system was functioning effectively. Compared with the empty-vector control (TRV2), the *SlLhcb13*-TRV2 silenced plants were more susceptible to gray mold, exhibiting larger lesions on detached leaves and pronounced wilting symptoms throughout the whole plant (Figure 8C–E). In the transient overexpression assay performed in *N. benthamiana*, the *SlLhcb13*-3FLAG-overexpressing plants exhibited enhanced resistance to gray mold compared with the empty-vector control, as evidenced by significantly reduced lesion areas (Figure 8G,H). To verify the gene expression levels in these materials, we performed qRT-PCR analysis. The results showed that the expression level of *SlLhcb13* was significantly reduced in the VIGS-silenced tomato plants (Figure 8F), whereas it was significantly increased in the overexpression lines of *N. benthamiana* (Figure 8I).

Collectively, silencing of *SlLhcb13* compromises resistance in tomato, while its overexpression enhances resistance in *N. benthamiana*. Taken together, clearly demonstrating that *SlLhcb13* functions as a key positive regulator of tomato resistance to *B. cinerea*.

## 3. Discussion

As a globally important vegetable crop and a model species within the Solanaceae, tomato has substantial theoretical and practical value for understanding disease resistance mechanisms. Although previous studies have demonstrated that the application of biochar, specific biopesticides, or exogenous hormones can effectively induce systemic resistance to gray mold in tomato [41,42,43,44], the intrinsic molecular basis of this resistance, particularly the post-transcriptional regulatory network, remains insufficiently characterized. In the present study, using tomato ‘Ailsa Craig’ as the experimental material, we integrated physiological, biochemical, transcriptomic, and proteomic analyses to dissect the regulatory mechanisms and molecular networks underlying the tomato response to gray mold.

Infection by *B. cinerea*, a typical necrotrophic pathogen, is commonly accompanied by a burst of host ROS and dynamic reprogramming of the antioxidant system. In this study, 24 h after inoculation with *B. cinerea*, SOD activity in tomato seedlings decreased significantly, whereas POD and CAT activities increased significantly. This pattern is consistent with the changes in antioxidant enzyme activities reported during the early interaction between tomato and *B. cinerea* [45,46], but differs from the continuously increasing SOD activity observed in chili, kiwifruit, and apple following *B. cinerea* infection [47,48,49]. These differences suggest that *B. cinerea* may interfere with the tomato SOD system, possibly through the secretion of specific effector proteins, although this hypothesis requires further validation. Infection with *B. cinerea* also caused marked increases in MDA and H_2_O_2_ contents in tomato, which were strongly associated with severe browning and wilting symptoms. This observation is consistent with the positive relationship between oxidative damage and phenotypic deterioration reported by Liang et al. [50], Zhang et al. [51] and Ma et al. [52] in studies of gray mold, further supporting the view that membrane lipid peroxidation is a critical step in *B. cinerea*-induced host cell death.

Integrated multi-omics analysis provides a multidimensional framework for elucidating plant disease resistance mechanisms. In this study, combined transcriptomic and proteomic analyses identified 5824 DEGs and 124 DEPs. Among these, only 100 genes showed significant changes at both the transcript and protein levels. This result is consistent with observations reported in studies of the *Arabidopsis* response to gray mold and the tomato response to early blight [53], highlighting the widespread occurrence of extensive post-transcriptional regulation during plant responses to gray mold. Particularly noteworthy was the identification of four genes showing opposite regulatory trends, which corresponds to the “transcription-protein expression decoupling” phenomenon described by Xu et al. [54] and Son et al. [55] in plant–pathogen interactions. This finding suggests the presence of refined regulatory mechanisms, such as microRNA-mediated translational repression or selective protein degradation, and provides new clues for future investigation of translational regulatory networks in plant immunity. Due to the limited sampling time points (0 h and 24 h) in this study, finer molecular fluctuations during the infection process were not captured. This is a limitation of the current study, and future research will include additional time points to further resolve these dynamic processes.

Functional enrichment analysis further revealed that the overlapping DEGs and DEPs were significantly enriched in defense-related pathways, including defense response to fungus, plant–pathogen interaction, and phenylpropanoid biosynthesis. These findings are consistent with well-known defense strategies in plant immunity. As a key module in plant–pathogen interaction pathways, the evolutionary conservation of MAPK cascades has been widely reported across different crop species [56,57]. Meanwhile, phenylpropanoid metabolites, especially flavonoid phytoalexins and lignin precursors that strengthen cell wall structure, have been well documented in studies of tomato disease resistance research [58]. Importantly, the present study verified activation of these pathways at the translational level through proteomic analysis, thereby providing more direct molecular evidence for the execution of defense responses than could be inferred from transcriptomic data alone.

Jasmonic acid (JA) and salicylic acid (SA) signaling pathways play pivotal roles in plant disease resistance. In this study, although key genes in the JA/SA pathways, such as *SlTomloxC*, *SlAOS*, *SlPDF1.2*, and *SlPR-1*, showed transcriptional changes, their corresponding protein levels remained largely unchanged. This discrepancy between transcript abundance and protein accumulation should not be interpreted as ineffective regulation; rather, it likely reflects a mechanism for fine control of signal transduction. By rapidly modulating transcript levels, plants may immediately perceive JA/SA-related signals, such as pathogen invasion, and thereby establish a transcriptional reserve for subsequent responses. At the same time, relatively stable protein levels may prevent unnecessary energy consumption or cytotoxic effects caused by excessive protein accumulation, while allowing dynamic modulation of signaling output through protein turnover, including synthesis and degradation. Such a mechanism would enable the JA/SA signaling pathways to respond rapidly while remaining under precise control, which is consistent with the classical concept of JA/SA-mediated resistance to necrotrophic pathogens [59].

Chloroplasts play a central role in plant immunity through the regulation of redox homeostasis and ROS [60,61,62]; however, the mechanisms by which the necrotrophic pathogen *B. cinerea* manipulates host chloroplast function remain unclear. Accumulating evidence indicates that specific LHC subclasses participate in disease resistance by finely tuning reactive oxygen species (ROS) homeostasis. For instance, phosphorylation of Lhcb proteins triggers broad-spectrum blast resistance in rice, whereas *CaLhcb1*-like coordinates ROS dynamics and lignin deposition to establish a defense barrier in pepper [39,63]. Although previous studies have shown that chloroplast-localized proteins, such as HHL1 and Lhcb5, can participate in disease resistance by regulating photosystem stability and ROS signaling [39,64], the specific roles of LHC family members in the tomato response to *B. cinerea* have not yet been fully elucidated. Through integrated multi-omics analysis combined with functional validation, the present study identified *SlLhcb13*, a member of the LHCB family, as a positive regulator of tomato resistance to *B. cinerea*. Notably, the transcript and protein levels of *SlLhcb13* showed uncoupled expression patterns after *B. cinerea* infection. Transcript abundance was significantly induced at 1 h post-inoculation but declined at 24 h, whereas protein accumulation increased at 24 h. This discrepancy suggests that *SlLhcb13* may be subject to a feedback regulatory mechanism. *SlLhcb13* may be rapidly transcribed during the early stage of infection to initiate defense responses, whereas sustained protein accumulation at later stages may trigger negative feedback that suppresses further transcription. Such temporal regulation may enable fine tuning of plant immunity while maintaining cellular homeostasis, consistent with the role of *SlLhcb13* as a positive regulator of tomato resistance to gray mold. While the VIGS data establish a necessary role for SlLhcb13 in tomato immunity, stable transgenic overexpression in tomato would provide definitive confirmation of sufficiency in the native host. This is noted as a valuable direction for future research. Mechanistically, *SlLhcb13* may function as a key node linking photosynthesis and defense by balancing photosynthetic efficiency with induced defense responses through regulation of energy allocation, modulation of ROS signaling, and control of defense-related gene expression. These findings provide a systematic framework for understanding the molecular basis of the tomato response to *B. cinerea* infection and suggest a potential role of the photosynthetic apparatus in plant immunity, thereby offering novel hypothetical target genes and theoretical insights for molecular breeding strategies aimed at enhancing crop disease resistance.

While the VIGS data establish a necessary role for *SlLhcb13* in tomato immunity, it is important to note that the overexpression assay was performed in the heterologous *N. benthamiana* system. Stable transgenic overexpression of *SlLhcb13* in tomato would provide definitive confirmation of its sufficiency in the native host and fully validate the causal relationship between this gene and gray mold resistance. This represents a valuable direction for future research to complement the current findings.

## 4. Materials and Methods

### 4.1. Plant Material and Growth Conditions

The tomato cultivar ‘Ailsa Craig’ was used as the experimental material. Plants were grown in a controlled growth chamber at the Horticultural Experimental Center of Shanxi Agricultural University. The growth conditions were set to a 16 h photoperiod (250 µmol·m^−2^·s^−1^), with temperatures of 25 °C during the day and 20 °C at night.

### 4.2. Cultivation and Inoculation of B. cinerea

*B. cinerea* cultivation and inoculation followed established protocols with minor modifications [65]. A mycelial plug of *B. cinerea* (strain *B05.10*) stored at −80 °C was retrieved and subcultured on PDA medium for activation. The culture plates were incubated at 20 ± 2 °C for 10–15 days until the mycelium had fully covered the plate surface. Spores were then washed from the plate surface with sterile water, and the suspension was filtered through two layers of Miracloth (Millipore, Billerica, MA, USA) to remove hyphal fragments, yielding a spore suspension. Spore concentration was determined using a hemocytometer under a microscope and adjusted to a final concentration of 1 × 10^5^ spores·mL^−1^ with potato dextrose broth (PDB) medium.

In vitro inoculation: Detached leaves were placed on filter paper moistened with sterile water. A 10 μL aliquot of the *B. cinerea* spore suspension was applied to both sides of the leaf midrib. The leaves were then transferred to sealed containers to maintain high humidity and incubated at 20 ± 2 °C and 90% relative humidity under a 16 h light/8 h dark photoperiod. Disease symptoms were assessed at 72 h post-inoculation, photographed, and lesion areas were measured. At least 20 leaves were used for each treatment, and three biological replicates were performed.

In planta inoculation: Healthy tomato seedlings showing uniform and vigorous growth were evenly sprayed with the spore suspension. After inoculation, the plants were placed in sealed containers to maintain humidity and then transferred to a growth chamber. The environmental conditions were the same as those used for in vitro leaf inoculation.

### 4.3. Assays for Antioxidant Enzyme Activities

#### 4.3.1. SOD, POD, and CAT Activity Determination

Tomato seedlings inoculated with *B. cinerea* (four true leaves, uniform growth stage) were sampled at 0, 24, and 48 h post-treatment, with three biological replicates for each time point. Leaf samples were immediately frozen in liquid nitrogen and stored at −80 °C until analysis. For enzyme extraction, 0.5 g of frozen leaf tissue was homogenized in 5 mL of ice-cold 50 mM phosphate buffer using a pre-chilled mortar. The homogenate was transferred to a 10 mL centrifuge tube and centrifuged at 12,000× *g* for 15 min at 4 °C. The supernatant was collected as the crude enzyme extract and kept on ice for subsequent analyses. SOD activity was measured using the nitroblue tetrazolium (NBT) photoreduction method, POD activity was determined by the guaiacol oxidation method, and CAT activity was assayed by monitoring the decomposition of H_2_O_2_ at 240 nm.

#### 4.3.2. MDA Content Determination

MDA content was determined using the thiobarbituric acid (TBA) colorimetric method. Leaf tissue (0.3 g) was homogenized in 5 mL of 5% (*w*/*v*) trichloroacetic acid and then centrifuged at 12,000× *g* for 15 min to obtain the supernatant. An equal volume of TBA reagent (containing trichloroacetic acid) was added to the supernatant, and the mixture was heated in a 95 °C water bath for 30 min. After cooling and a second centrifugation step, the supernatant was collected, and absorbance was measured at 532 nm. Blank and standard samples were included, and MDA content was calculated from a standard curve with correction for dilution factors.

#### 4.3.3. H_2_O_2_ Content Determination

H_2_O_2_ content was measured by ammonium molybdate colorimetry. Frozen leaf powder (2 g) was homogenized in 5 mL of pre-chilled acetone and then centrifuged at 1000× *g* for 20 min at 4 °C. The resulting supernatant was mixed with sulfuric acid-acidified ammonium molybdate reagent and allowed to react at room temperature for 15 min. Absorbance was then measured at 405 nm. Blank and standard samples were included, and H_2_O_2_ content was calculated from a standard curve with correction for dilution and sample volume.

### 4.4. Transcriptome Analysis

When tomato plants reached the four-leaf stage, they were inoculated with a spore suspension (1 × 10^5^ spores·mL^−1^), and leaf samples were collected at 0 and 24 h post-inoculation. Three biological replicates were included for each treatment. After rapid freezing in liquid nitrogen, high-quality total RNA was extracted for strand-specific mRNA library construction. Transcriptome sequencing was performed on the MGISEQ platform. Raw reads were quality filtered, trimmed, and mapped to the tomato reference genome (SL4.0). Differentially expressed genes (DEGs) were identified using DESeq2 with the criteria |log_2_FC| > 1 and adjusted *p*-value (FDR) < 0.05. ClusterProfiler was used for Gene Ontology (GO) functional annotation and KEGG pathway enrichment analysis of DEGs to elucidate the biological pathways activated during *B. cinerea* infection. Transcriptome analysis was conducted following standard RNA-seq protocols [66].

### 4.5. Proteomics Analysis

Total proteins were extracted from the same leaf samples used for transcriptome analysis, and protein concentrations were determined by the Bradford assay. Protein extracts were reduced with 10 mM dithiothreitol (DTT) at 56 °C for 1 h and then alkylated with 55 mM iodoacetamide (IAM) in the dark for 45 min. Trypsin was subsequently added at an enzyme-to-substrate ratio of 1:50 (*w*/*w*), and digestion was carried out at 37 °C for 4 h. After desalting, the peptides were labeled with TMT (Tandem Mass Tag) reagents. The labeled peptides were separated using an Easy-nLC 1200 nanoflow liquid chromatography system coupled to an Orbitrap Exploris 480 mass spectrometer for data-dependent acquisition (DDA). Differentially expressed proteins (DEPs) were identified using the criteria |log_2_FC| > 0.58 (equivalent to fold change > 1.5) and FDR < 0.05. Proteomic analysis was performed based on established mass spectrometry protocols [67].

### 4.6. Construction of Overexpression Vector

Based on the CDS sequence of *SlLhcb13*, full-length primers containing the start codon, stop codon, and homologous recombination arms were designed. The overexpression vector pGATE8-*SlLhcb13*-3FLAG was constructed by homologous recombination cloning. The pHELLSGATE8 empty vector and pGATE8-*SlLhcb13*-3FLAG were transformed into *Agrobacterium tumefaciens* GV3101, followed by small-scale culture and large-scale propagation. The bacterial culture was grown to an OD_600_ of 0.8, centrifuged at 3500× *g* for 5 min at room temperature, and the pellet was resuspended in infiltration buffer (10 mM MgCl_2_, 10 mM MES, pH 5.6, 200 μM acetosyringone) to an OD_600_ of 0.5. After incubation in the dark for 2–3 h, the suspension was infiltrated into *Nicotiana benthamiana* leaves. The infiltrated leaves were maintained in the dark for 24 h and then returned to normal light conditions. After 2 days, the leaves were harvested for *B. cinerea* inoculation. Disease symptoms and lesion areas were recorded at 72 h post-inoculation, and gene expression was analyzed by qRT-PCR. All primers used are listed in Supplementary Table S1.

### 4.7. Construction of VIGS Silencing Vector

The SGN VIGS Tool (https://vigs.solgenomics.net/ (accessed on 10 March 2025)) was used to design specific VIGS fragments for *SlLhcb13*. Target fragments flanked by homologous arms were synthesized and cloned into the pTRV2 vector by homologous recombination to generate pTRV2-*SlLhcb13*. *Agrobacterium* strains carrying pTRV1, pTRV2, pTRV2-*SlLhcb13*, and pTRV2-PDS were streaked on selective medium, subjected to small-scale culture followed by large-scale propagation, and grown to an OD_600_ of 0.8–1.0. The cultures were centrifuged at 5000× *g* for 10 min, and the bacterial pellets were resuspended in infiltration buffer to an OD_600_ of 1.0. After incubation at room temperature for 2–3 h, pTRV1 was mixed in equal volumes with pTRV2, pTRV2-*SlLhcb13*, and pTRV2-PDS and then infiltrated into tomato seedlings at the two-leaf stage. After infiltration, the plants were kept in the dark for 24 h and then transferred to normal light conditions. When the photobleaching phenotype appeared (10–15 days post-infiltration), gene silencing efficiency was evaluated by qRT-PCR. Systemic leaves (the second and third leaves below the shoot apical meristem) were subsequently collected for *B. cinerea* inoculation. Disease symptoms were assessed at 72 h post-inoculation, and lesion areas were recorded. All primers used are listed in Supplementary Table S1.

### 4.8. Statistical Analysis

Data were processed using Microsoft Excel. Lesion areas were quantified with ImageJ 1.52. Statistical analyses were performed using GraphPad Prism 8.0, and significance was determined by one-way ANOVA followed by Duncan’s multiple range test (*p* < 0.05).

## 5. Conclusions

This study systematically elucidates the physiological mechanisms and molecular regulatory networks underlying tomato response to *B. cinerea*. Physiological analyses showed that infection by *B. cinerea* (gray mold) triggered a series of time-dependent oxidative stress responses, characterized by significant alterations in the antioxidant enzyme system and membrane lipid peroxidation products. These results indicate that tomato activates specific defense mechanisms in response to pathogenic stress. Integrated transcriptomic and proteomic analyses identified 5824 differentially expressed genes and 124 differentially expressed proteins, which were mainly enriched in pathways related to plant–pathogen interaction, metabolism, and photosynthesis. Notably, the discordance between transcriptomic and proteomic profiles highlighted the central role of post-transcriptional regulation in stress adaptation. Further analysis focused on a key photosynthesis-related gene, *SlLhcb13*. Although *SlLhcb13* was transcriptionally suppressed during infection, its encoded chlorophyll-binding protein was significantly upregulated at the translational level. This post-transcriptional regulatory pattern was validated by qRT-PCR and transient transformation assays. Functional characterization further confirmed that *SlLhcb13* positively regulates tomato resistance to *B. cinerea*, serving a dual role in maintaining photosynthetic efficiency and mediating defense responses. Collectively, these findings not only expand the theoretical understanding of plant resistance to gray mold but also provide valuable candidate targets and theoretical support for molecular breeding aimed at improving disease resistance in crops.

## Data Availability

The raw RNA-seq data and TMT proteomics data generated in this study have been deposited to the NCBI Sequence Read Archive (SRA, accession number: SRP694086) and iProX (accession number: PXD077458), respectively.

## Supplementary Materials

The following supporting information can be downloaded at: https://www.mdpi.com/article/10.3390/plants15091360/s1, Figure S1: Clustering and subcellular localization of differentially expressed proteins; Figure S2: Correlation analysis between differentially expressed genes and proteins; Table S1: List of primers used in this study.
