# Supplementary material for: Multi-Omics Analysis Identifies SlLhcb13 as a Key Regulator of Tomato Resistance to Botrytis cinerea
Source: Plants (Basel). 2026 Apr 29;15(9):1360. doi: 10.3390/plants15091360 (PMC13165056; doi:10.3390/plants15091360)
Supplement: Supplementary file 1 [file plants-15-01360-s001.zip › plants-4261806-supplementary.pdf]

Supplementary Data

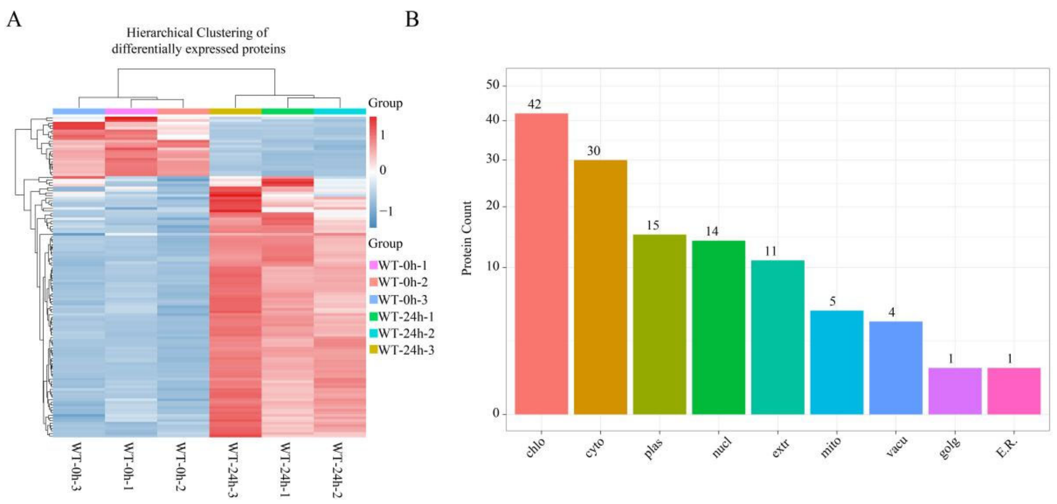

Figure S1. Clustering and subcellular localization of differentially expressed proteins.

(A) Hierarchical clustering heatmap of differentially expressed proteins. Red indicates upregulated proteins, whereas blue indicates downregulated proteins. Samples were clustered according to treatment conditions. (B) Subcellular localization distribution of differentially expressed proteins. The height of each bar indicates the number of differential proteins in each subcellular compartment.

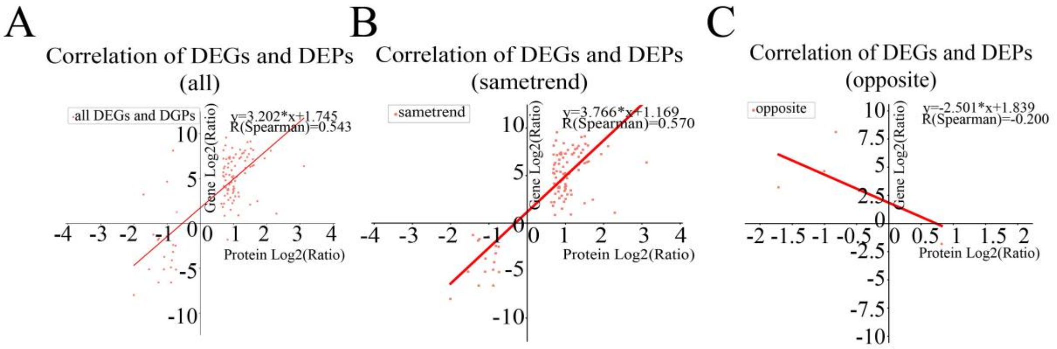

Figure S2. Correlation analysis between differentially expressed genes and proteins.

(A) Expression correlation of all significantly differentially expressed proteins and genes. (B) Expression correlation of proteins and genes showing consistent expression trends. (C) Expression correlation of proteins and genes showing opposite expression trends. The x-axis represents protein expression levels, and the y-axis represents gene expression levels.

Table S1. List of primers used in this study.

Primer name	Primer sequence (5'-3')
Q-Actin-FW	GTCCTCTTCCAGCCATCCAT
Q-Actin-RV	ACCACTGAGCACAATGTTACCG
Lhcb13-qPCR-FW	CAGTGACGGTGGACTTGACT
Lhcb13-qPCR-RV	CTTCTCCAACGCCTGGAAGT
35S-FW	ACGCACAATCCCACTATCCTTC
Lhcb13-3FLAG-FW	GATGACGATGACAAGGAATTCATGGCATCAATGGCAGCAAC
Lhcb13-3FLAG-RV	GTCCTTGTAATCCATGAATTCAGCTCCAGGAACAAACTTAG
Lhcb13-TRV2-FW	CTGTGAGTAAGGTTACCGAATTCAAGATGGGCAATGCTTGG
Lhcb13-TRV2-RV	CGCGTGAGCTCGGTACCGGATCCCGGGGTATAAGTCGTTGC
HR-TRV2-FW	TTGTTACTCAAGGAAGCACGATG
HR-TRV2-RV	CCTAAAACTTCAGACACGGATCTACT
